# Chemical and Enantioselective Analysis of the Leaf Essential Oil from *Varronia crenata* Ruiz & Pav. Growing in Ecuador

**DOI:** 10.3390/molecules31030532

**Published:** 2026-02-03

**Authors:** Karem Cazares, Yessenia E. Maldonado, Nixon Cumbicus, Gianluca Gilardoni, Omar Malagón

**Affiliations:** 1 Departamento de Química, Universidad Técnica Particular de Loja (UTPL), Calle París s/n y Praga, Loja 110107, Ecuador; kcazares@uea.edu.ec (K.C.); or ggilardoni@utpl.edu.ec (G.G.); 2 Departamento de Matemáticas y Ciencias Físicas, Universidad Estatal Amazónica, Puyo 160150, Pastaza, Ecuador; 3 Maestría en Ciencias Químicas, Escuela Superior Politécnica de Chimborazo (ESPOCH), Panamericana Sur km 1 1/2, Riobamba 060155, Ecuador; 4 Programa de Doctorado en Química, Universidad Técnica Particular de Loja (UTPL), Calle París s/n y Praga, Loja 110107, Ecuador; yemaldonado2@utpl.edu.ec; 5 Departamento de Ciencias Biológicas y Agropecuarias, Universidad Técnica Particular de Loja (UTPL), Calle París s/n y Praga, Loja 110107, Ecuador; nlcumbicus@utpl.edu.ec

**Keywords:** *Varronia crenata*, essential oil, enantiomers, chiral separation

## Abstract

Essential oils from species of the genus *Varronia* (Boraginaceae) are recognized for their chemical diversity and biological potential; however, phytochemical information on *Varronia crenata* Ruiz & Pav. remains scarce, despite its wide distribution in the Andean region. The aim of this study was to provide the first chemical and enantioselective characterization of the essential oil obtained from the leaves of *V. crenata* growing in Ecuador. Qualitative and quantitative analyses were carried out by GC–MS and GC–FID, respectively, using two columns with stationary phases of contrasting polarity. Compounds were identified by matching linear retention indices and mass spectra to literature references and quantified by external calibration using relative response factors (RRFs) calculated for each compound based on its combustion enthalpy. The most abundant constituents (≥3.0% on average between the two columns) of the essential oil of *V. crenata*, both in the nonpolar and polar stationary phases, were germacrene D (18.4%), *(E)*-β-caryophyllene (13.3%), α-copaene (10.4%), tricyclene (9.3%), δ-cadinene (8.9%), and α-pinene (8.3%). The volatile fraction was dominated by sesquiterpenes (60.2%) and monoterpenes (22.1%), while other chemical families were present in minor proportions. The enantioselective analysis was performed on two different columns, coated with stationary phases based on β-cyclodextrins: 2,3-diacetyl-6-*tert*-butyl-dimethylsilyl-β-cyclodextrin and 2,3-diethyl-6-*tert*-butyl-dimethylsilyl-β-cyclodextrin. Nine chiral compounds were analyzed; among them, *(1R,5R)*-(+)-α-pinene, *(1R,5R)*-(+)-sabinene, and *(S)*-(+)-β-phellandrene were detected as enantiomerically pure, while the other metabolites presented scalemic mixtures. Overall, the high content of bioactive sesquiterpenes and the observed stereochemical complexity highlight the potential pharmaceutical and agricultural relevance of *V. crenata* essential oil, while also providing novel chemotaxonomic information for the genus.

## 1. Introduction

Since ancient times, aromatic and medicinal plants have been prized for their healing and fragrant properties, making them an important source of bioactive compounds with therapeutic, cosmetic, and food applications [1]. With the development of natural product chemistry, it has been proven that these properties are due to their complex composition of secondary metabolites, mainly terpenes and phenylpropanoids, which are responsible for most of their antimicrobial, anti-inflammatory, antioxidant, and cytotoxic effects [2]. In the field of phytochemistry, essential oils (EOs) stand out. These are complex mixtures of volatile compounds synthesized and stored in different specialized secretory structures, such as glandular trichomes, cavities, or oil ducts, where highly volatile metabolites are concentrated [3].

Ecuador is recognized by the United Nations Environment Programme’s World Conservation Monitoring Center as one of the 17 megadiverse countries in the world. It has a wide variety of species, many of which are unique to the country. In terms of flora, Ecuador has more than 5000 endemic species [4]. Despite this, phytochemical research on many native species remains limited in the country [5,6]. For this reason, the authors have been researching the secondary metabolites present in Ecuadorian flora for more than 20 years, with the aim of expanding knowledge in the fields of natural product chemistry and phytopharmacology.

Boraginaceae taxa have been traditionally used for centuries in different cultural contexts, and their ethnopharmacological relevance has been progressively supported by phytochemical and biological studies. Members of this family are known to produce a wide variety of secondary metabolites, including fatty acids, essential oils, phenolic compounds, flavonoids, naphthoquinones, and other bioactive constituents, which account for their therapeutic and functional properties [7]. Extracts from Boraginaceae species have been associated with anti-inflammatory, antimicrobial, antioxidant, and skin-protective effects, supporting their application in pharmaceutical, cosmetic, and related biotechnological fields [8,9].

In this context, the species of genus *Varronia* (Boraginaceae) have attracted growing scientific interest, due to the wide range of secondary metabolites they produce, including monoterpenes, sesquiterpenes, and phenolic compounds, that contribute significantly to their chemical profile and biological potential [10]. This neotropical genus comprises more than 125 species recorded to date. Most of them are distributed in the American continent in countries such as Brazil, Mexico, and the northern Andes region, where this plant group has a strong presence [11]. These species are distinguished by their multi-stemmed shrub habit, serrated leaves, condensed inflorescences, and triporate pollen grains with a reticulate tectum [12].

Among the species belonging to this family, *Varronia crenata* Ruiz & Pav. is the subject of the present study. It is an aromatic shrub species, distributed across Ecuador, Peru and Bolivia. According to Tropicos, *V. crenata* is also known under two widely used synonyms: *Cordia lantanoides* Spreng. and *Varronia rusbyi* (Britton ex Rusby) Borhidi [13]. However, a review of Plants of the World Online indicates that *V. rusbyi* is treated as a synonym of *Cordia alliodora*. Our voucher specimen, and its comparison with reference material for *C. alliodora*, do not match that taxon [14]. Known locally as “wakra kallu” (Kichwa), “sacha ortiga” (Spanish–Kichwa), “matico”, “morochillo”, and “yanango”, *V. crenata* (reported in the cited literature under the synonym *C. lantanoides*) is traditionally used by Andean communities as food and medicine, and as a source of fuel [15]. This plant grows at altitudes between 2000 and 3000 m above sea level, where it inhabits montane forests and Andean scrublands, characterized by persistent fog and a temperate humid climate [13,14,15,16,17]. Despite its wide distribution and traditional use, especially in medicine to treat wounds [15], phytochemical information on this species is practically non-existent. Previous studies on related species such as *V. curassavica* [18,19,20,21], *V. multispicata* [22,23], and *V. globosa* [24] have revealed essential oils and non-volatile fractions rich in oxygenated sesquiterpenes, monoterpenes, and phenolic compounds with important antioxidant, antibacterial, and anti-inflammatory properties [25,26,27,28,29]. In view of the above, the species *V. crenata* was selected to study the chemical and enantiomeric composition of its essential oil. The purpose of this work is to expand knowledge about the volatile metabolites of Ecuadorian flora, as well as to identify compounds of interest for pharmaceutical, cosmetic, and biotechnological applications, while promoting the sustainable valorization of the country’s native plant resources.

## 2. Results

### 2.1. Chemical Composition of the EO

The dried leaves of *V. crenata* produced an EO with a distillation yield of 0.08 ± 0.006%. Chemical analysis was performed using two columns with stationary phases of different polarity, one nonpolar (5% phenyl methyl polysiloxane) and one polar (polyethylene glycol). A total of 46 compounds were detected, most of them (45) were identified by comparing their electron impact mass spectra (EIMS) and linear retention indices (LRI) with data reported in the literature, while one remained unidentified. Based on its molecular weight (236 amu), the unidentified compound probably corresponded to a dioxygenated sesquiterpenoid. The total amount of quantified compounds represented 87.2–85.6% of the total EO mass on the nonpolar and polar stationary phases. The volatile fraction was mainly composed of sesquiterpene hydrocarbons (60.4–60.0%), followed by a smaller proportion of monoterpenes (22.0–22.1%), while the other chemical groups only represented about 2.2–1.8%. The dominant constituents (≥3.0% in at least one column) were germacrene D (18.7–18.1%, **35**), *(E)*-β-caryophyllene (13.3–13.3%, **29**), α-copaene (10.2–10.5%, **25**), tricyclene (9.4–9.1%, **1**), δ-cadinene (8.9–8.8%, **40**), and α-pinene (8.2–8.4%, **2**). Together, these abundant metabolites defined the characteristic chemical profile of *V. crenata* EO, which reflected a remarkable biochemical complexity, as well as significant potential as a natural source of bioactive compounds.

**Figure 1:**
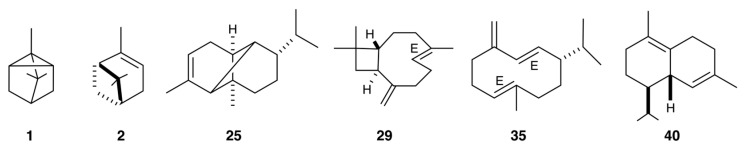
Major components (≥3.0% in at least one column) of *V. crenata* leaf EO. The numbers refer to Table 1: tricyclene (**1**), α-pinene (**2**), α-copaene (**25**), *(E)*-β-caryophyllene (**29**), germacrene D (**35**), and δ-cadinene (**40**).

**Figure 2:**
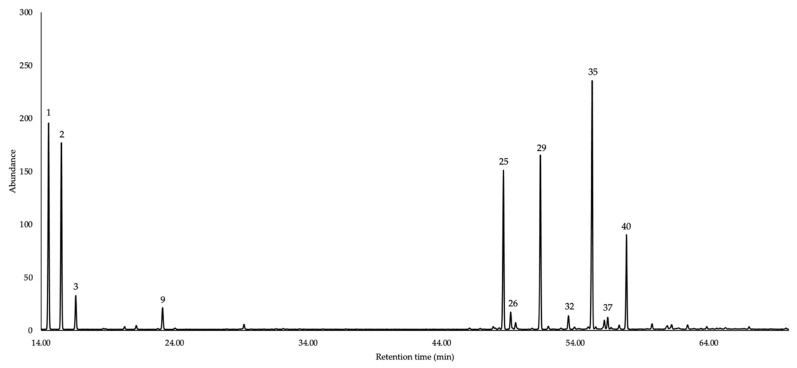
GC-MS profile of *V. crenata* EO using a 5% phenyl methyl polysiloxane stationary phase. Peak numbers correspond to the major constituents (≥3.0% in at least one column) listed in Table 1.

**Figure 3:**
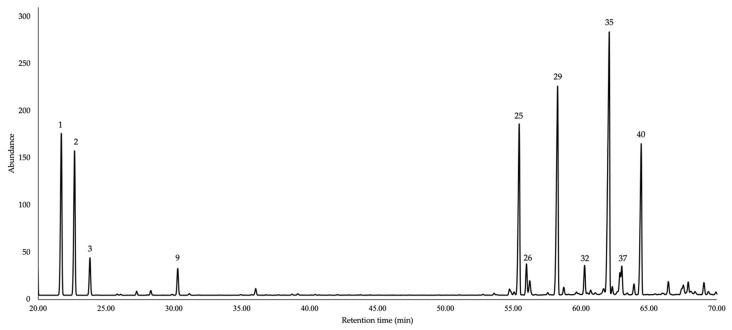
GC-MS profile of *V. crenata* EO using a polyethylene glycol stationary phase. Peak numbers correspond to the major constituents (≥3.0% in at least one column) reported in Table 1.

The chemical structures of the main components of this EO are shown in Figure 1, while the gas chromatographic (GC) profiles are presented in Figure 2 and Figure 3. The complete results of the qualitative and quantitative analyses are detailed in Table 1.

### 2.2. Enantioselective Analysis

The enantioselective analysis of *V. crenata* EO allowed the identification of nine chiral metabolites, corresponding to both monoterpene and sesquiterpene hydrocarbons. Separation was performed using two columns whose chiral selector was based on β-cyclodextrins: 2,3-diacetyl-6-*tert*-butyl-dimethylsilyl-β-cyclodextrin (DAC) and 2,3-diethyl-6-*tert*-butyl-dimethylsilyl-β-cyclodextrin (DET), selected for their differentiated chiral resolution properties. The detailed results of the enantioselective analyses are presented in Table 2.

Among the analyzed chiral compounds, *(1R,5R)*-(+)-α-pinene, *(1R,5R)*-(+)-sabinene, and *(S)*-(+)-β-phellandrene were detected as enantiomerically pure, indicating highly stereospecific biosynthesis for these monoterpenes. The other identified chiral compounds were present as scalemic mixtures, where *(S)*-(+)-α-phellandrene showed a high enantiomeric excess (greater than 90%). In contrast, linalool showed an almost racemic distribution.

## 3. Discussion

The essential oil yield obtained from *Varronia crenata* leaves (0.08% *w*/*w*) is low; however, low or extremely low yields have been reported for other species of the genus *Varronia* and related Boraginaceae taxa. For instance, *Varronia globosa* [24], *Cordia verbenacea* D.C. [25], *Cordia curassavica* [63], and *Borago officinalis* L. [64,65] have been described as producing low essential oil yields, often below 1% based on dry weight.

**Figure 4:**
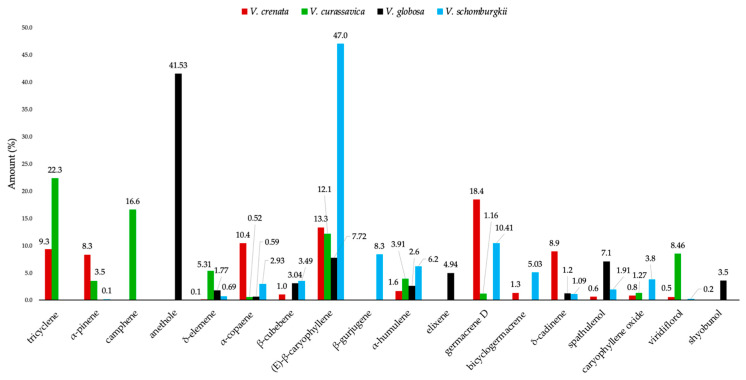
Comparative abundance of major constituents (≥3.0% in at least one oil) in the leaf EOs of *V. crenata* (red), *V. curassavica* (green) [29], *V. globosa* (black) [24], and *V. schomburgkii* (cyan) [66].

When comparing the chemical composition of the EO of *V. crenata* with the one reported for other species of the genus *Varronia*, notably different profiles are evident [24,29,66]. Figure 4 shows the comparison of the main components (≥3.0% in at least one of the oils) identified in these species.

The essential oil of *V. schomburgkii* had a typical sesquiterpene composition, clearly dominated by *(E)*-β-caryophyllene (47.0%), accompanied by considerable proportions of germacrene D (10.4%), β-gurjunene (8.3%), α-humulene (6.2%), bicyclogermacrene (5.0%), β-cubebene (3.5%), and caryophyllene oxide (3.75%). This sesquiterpene profile reflected a characteristic feature of the Boraginaceae family [67]. On the other hand, *V. globosa* showed a volatile fraction clearly dominated by anethole (41.5%), a phenylpropanoid responsible for the sweet, aniseed aroma of the oil, reflecting the involvement of the shikimic acid pathway [68]. Considerable proportions of *(E)*-β-caryophyllene (7.7%), spathulenol (7.1%), elixene (4.9%), shyobunol (3.5%), and β-cubebene (3.0%) evidenced the coexistence of metabolites derived from different biosynthetic pathways.

In the case of *V. curassavica*, the EO showed a mixed profile of monoterpenes and sesquiterpenes, with tricyclene (22.3%), camphene (16.6%), α-pinene (3.5%), δ-elemene (5.3%), *(E)*-β-caryophyllene (12.1%), α-humulene (3.9%), viridiflorol (8.5%), α-pinene (16.6%), and germacrene D (10.4%) as major components. Notably, *(E)*-β-caryophyllene was present in all four analyzed species, although in varying concentrations. Interspecific variability is mainly due to genetic differences between species of the same genus, which determine particular biosynthetic pathways and enzymes responsible to produce secondary metabolites [69]. In addition, to some extent, diversity could be influenced by geographical and bioclimatic factors, that regulated the activity of enzymes involved in the biosynthesis of terpenoids and phenylpropanoids [70,71].

The main compounds identified in the EO of *V. crenata*, such as *(E)*-β-caryophyllene, tricyclene, α-copaene, α-pinene, δ-cadinene, and germacrene D, are recognized for their biological activities. For instance, *(E)*-β-caryophyllene (**29**), a bicyclic sesquiterpene, stood out for its anti-inflammatory, antioxidant, neuroprotective, and antitumor potential, attributed to its selective interaction with the CB2 receptor and the inhibition of proinflammatory mediators such as NF-κB and COX-2 [72,73,74]. Tricyclene (**1**), a monoterpene, has exhibited antioxidant, antimicrobial, and antitumor potential, as well as energetic properties as a precursor for biofuels, due to its compact tricyclic structure [75,76]. The sesquiterpene α-copaene (**25**) showed antimicrobial and antioxidant properties, together with an attractive effect on fruit insects, suggesting possible applications in biocontrol and food preservation [77,78,79]. The monoterpene α-pinene (**2**) demonstrated a wide range of biological effects, including antimicrobial, antifungal, antiparasitic, and neuroprotective activity, associated with the modulation of inflammatory and antioxidant pathways [80]. After that, δ-cadinene (**40**) exhibited antimicrobial and immunomodulatory properties, promoting the activation of neutrophils and the regulation of inflammatory processes [81,82]. Finally, germacrene D, the major compound, was associated with ecological functions such as attracting pollinating insects, acting as a kairomone and reinforcing its relevance in plant biocommunication [83,84].

**Figure 5:**
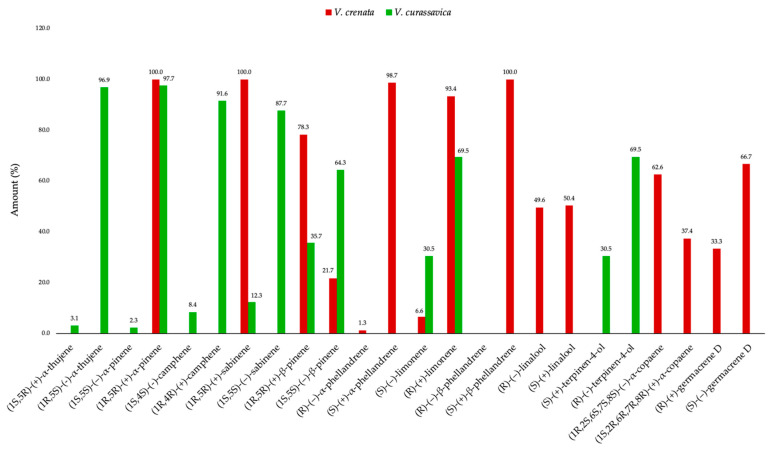
Comparative enantiomeric composition of selected chiral compounds in the leaf EOs of *V. crenata* (red) and *V. curassavica* (green) [85].

Due to the lack of enantioselective studies on the EOs from this genus, the enantiomeric composition of *V. crenata* EO can only be compared, within the same taxon, with the one of *V. curassavica* [85]. The results of this comparison are presented in Figure 5.

With this regard, no common pattern was found among the two species, where only α-pinene showed a similar enantiomeric distribution. Furthermore, an interesting phenomenon was observed in *V. crenata* EO: certain chiral compounds, which share the same chiral precursor in their biosynthetic pathway, exhibited unexpected enantiomeric distributions. This is the case with α-pinene and β-pinene, both derived from the pinyl cation, from which they acquire the configuration of their stereogenic centers [86]. Therefore, it would be reasonable to expect them to have a similar enantiomeric distribution, with comparable enantiomeric excesses. However, the results showed that *(1R,5R)*-(+)-α-pinene is enantiomerically pure, while β-pinene corresponds to a scalemic mixture, with an enantiomeric excess towards its dextrorotatory form. Two hypotheses could be formulated to explain this phenomenon: (1) a post-synthetic enantiospecific reactions could have occurred on the laevorotatory form of α-pinene, completely consuming it; (2) a partial racemisation has occurred during steam-distillation on the enantiomerically pure β-pinene. Racemisation is especially important in alcohols, such as linalool, where high temperature and acidic pH conditions favor the formation of allylic carbocations, capable of causing configuration inversion at the stereogenic center.

Distillation-induced racemization has been mainly reported for oxygenated monoterpenes, whereas hydrocarbon monoterpenes such as α-pinene and β-pinene are generally more stable. Therefore, β-pinene is not considered more susceptible to racemization than α-pinene, and its scalemic distribution in *Varronia crenata* is more likely related to biosynthetic stereochemical variability. This physicochemical phenomenon alters the natural stereochemical distribution of volatile metabolites and reduces their optical purity [87,88]. From a biochemical perspective, even small variations in the proportion of enantiomers can modify the affinity of compounds for receptors or enzymes, altering their biological and pharmacological activities, so that the interpretation of enantioselective results must be carried out with caution [89]. In the particular case pinenes, their biological activities are enantiospecific. The optical forms *(1R,5R)*-(+)-α-pinene and *(1R,5R)*-(+)-β-pinene show more marked antimicrobial activity against Gram-positive bacteria and phytopathogenic fungi, while their laevorotatory enantiomers exhibit lower efficacy or even no effect [90]. These variations are due to the stereochemical interaction between chiral monoterpenes and the active sites of microbial enzymes, that determines different biological activities for the enantiomers [91]. Likely, α-copaene exhibits clearly enantiospecific biological activities, where *(1S,2R,6R,7R,8R)*-(+)-α-copaene acts as a pheromone and sex attractant in *Ceratitis capitata* and stimulates oviposition in *Bactrocera oleae*, promoting insect-plant interaction [92,93]. In contrast, *(1R,2S,6S,7S,8S)*-(–)-α-copaene functions as a kairomone in xylophagous beetles, especially in *Euwallacea nr. fornicatus*, showing synergy with quercivorol [94]. Finally, it is pertinent to mention some considerations regarding the properties of *(S)*-(-)-germacrene D.

According to the scientific literature, the biological properties of germacrene D have not yet been fully explored; however, it has been shown that its laevorotatory form acts as a semiochemical, generating an attraction effect towards mated females of the moth *Heliothis virescens* and stimulating their oviposition.

Likewise, specific receptor neurons for *(S)*-(-)-germacrene D have been identified in other insects of the same genus, such as *H. armigera* and *H. assulta* [83,84].

## 4. Materials and Methods

### 4.1. Plant Material

The leaves of *V. crenata* were collected on 13 December 2023, at the slopes of mount Villonaco, at an altitude of 2610 m above sea level, in the province of Loja, Ecuador. The plant material was collected from different shrubs, within the range of 200 m from a central point of coordinates 4°00′10″ S and 79°15′3″ W. The leaves were reunited in a single mean sample, that was dried at 35 °C for 48 h the same day of collection. The plant collection was carried out under permit MAATE-DBI-CM-2022-0248, issued by the Ministry of Environment, Water, and Ecological Transition (MAATE). The species was morphologically identified by one of the authors (N.C.) and a voucher specimen was deposited in the herbarium of the Universidad Técnica Particular de Loja, under accession number HUTPL 15794.

### 4.2. Distillation and Sample Preparation

The dried leaves of *V. crenata* underwent analytical distillation, following the procedure previously described in the literature [95], in the same modified Dean–Stark apparatus. The whole amount of plant material was divided into four portions (38.0 g each), that were distilled, for four hours each, over 2 mL of analytical grade cyclohexane, containing n-nonane (1.4 mg) as an internal standard. After hydrodistillation, the cyclohexane layer containing the EO and n-nonane (internal standard) was separated from the aqueous phase and directly injected into the GC without solvent evaporation. The procedure was performed in quadruplicate (38.0 g plant material each; 2 mL cyclohexane; 4 h), and the resulting solutions were stored at −15 °C until analysis. Both cyclohexane and n-nonane were of analytical grade and obtained from Merck (Sigma-Aldrich, St. Louis, MO, USA).

### 4.3. Qualitative (GC-MS) Chemical Analyses

The qualitative analysis of *V. crenata* EO was performed using gas chromatography coupled with mass spectrometry (GC–MS), through a GC model Trace 1310 coupled with a single quadrupole mass spectrometer model ISQ 7000, both manufactured by Thermo Fisher Scientific (Waltham, MA, USA). The detection system operated in electron impact (EI) ionization mode at 70 eV, with the transfer line and ionization source set at 230 °C. The analysis was performed in SCAN mode, with a mass scan range between 40 and 400 *m/z*, under vacuum pressure. The carrier gas used was high-purity helium, supplied by Indura S.A. (Guayaquil, Ecuador), at a constant flow of 1 mL/min. Injections were performed in split mode (40:1) with the injector maintained at 230 °C. Two capillary columns with stationary phases of different polarity were used, which allowed for optimized separation and increased reliability in the identification of analytes. Two capillary columns with stationary phases of different polarity were used: 5% phenyl-methyl-polysiloxane (TR-5 ms, non-polar) and polyethylene glycol (TR-Wax, polar), both supplied by Thermo Fisher Scientific (Waltham, MA, USA).

Both columns had the following dimensions: 30 m in length, 0.25 mm in internal diameter, and 0.25 μm in film thickness. The oven temperature program started at 50 °C for 10 min, followed by an increase of 2 °C/min until reaching 170 °C, and then 10 °C/min until 230 °C, a temperature that was maintained for 20 min, with a total analysis duration of 96 min. For the TR-Wax column, the same thermal program was applied, varying only the injector temperature, which was set to 220 °C. The components were identified by comparing the mass spectra (MS) and linear retention indices (LRIs) calculated according to the methodology of Van den Dool and Kratz [96], with the values reported in the literature, using a series of C_9_–C_24_ n-alkanes. The alkanes used were provided by Merck (Sigma-Aldrich, St. Louis, MO, USA).

### 4.4. Quantitative (GC-FID) Chemical Analyses

The quantitative analysis of the essential oil was performed using the same gas chromatography system described for the qualitative analysis, configured with a flame ionization detector (FID). The operating conditions were identical to those used in the GC–MS, both for the non-polar and polar columns. The FID was fed with a gas mixture composed of hydrogen (35 mL/min) and air (350 mL/min), maintaining a final temperature of 230 °C for the non-polar column and 220 °C for the polar one. The carrier gas was helium, with a constant flow of 1 mL/min. The quantification of the analytes was performed calculating a relative response factor (RRF) for each compound, based on the combustion enthalpy, according to the mathematical model proposed by Alain Chaintreau [97,98]. The integration areas, once adjusted with the relative response factors (RRFs), were used to calculate two six-point calibration curves for each of the analytical columns. In these curves, n-nonane was used as the internal standard and isopropyl caproate as the calibration standard. The internal standard was provided by Merck (Sigma-Aldrich, St. Louis, MO, USA), while the calibration standard was synthesized by the authors in their laboratory and purified to a purity of 98.8%, determined by GC-FID. Both calibration curves recorded coefficients of determination (R^2^) greater than 0.998, and the standard solutions were prepared according to the procedures previously described in the literature [99].

### 4.5. Enantioselective Analyses

The enantioselective analysis of *V. crenata* EO was performed using gas chromatography coupled with mass spectrometry (GC–MS), employing the same MS conditions described in the qualitative analysis. Two enantioselective capillary columns were used to separate the enantiomers, whose stationary phases were based on β-cyclodextrin derivatives: 2,3-diacetyl-6-*tert*-butyl-dimethylsilyl-β-cyclodextrin (DAC) and 2,3-diethyl-6-*tert*-butyl-dimethylsilyl-β-cyclodextrin (DET), both from Mega S.r.l. (Legnano, Italy). The columns were 25 m long, with an internal diameter of 0.25 mm and a film thickness of 0.25 µm. The carrier gas was helium, maintained at a constant pressure of 75 kPa. The oven temperature program started at 50 °C, held for 5 min, followed by a ramp of 2 °C/min to 200 °C, which was maintained for 15 min. The injector and transfer line were set at 220 °C, and the spectrometer ionization source was maintained at 230 °C, with an ionization energy of 70 eV. The enantiomers were identified by comparing their LRIs and mass spectra with those of enantiomerically pure standards, some of them purchased from Merck (Sigma-Aldrich, St. Louis, MO, USA), while others were available from the University of Turin (Italy).

Since the mass spectra of two enantiomers are identical, the assignment of each optical configuration was performed exclusively by matching the LRIs with those of the standards. The LRI calculations were performed according to the Van den Dool and Kratz method [92] described in Section 4.3, using a homologous series of n-alkanes (C_9_–C_24_), injected under the same chromatographic conditions. The use of two different chiral selectors was due to the different separation capacities of each stationary phase for each enantiomeric pair. For example, the enantiomers of α-pinene were separated more effectively using 2,3-diacetyl-6-*tert*-butyl-dimethylsilyl-β-cyclodextrin, while the optical isomers of limonene and germacrene D showed better resolution using 2,3-diethyl-6-*tert*-butyl-dimethylsilyl-β-cyclodextrin.

### 4.6. Limitations

The current study mainly deals with the metabolic aspects of *V. crenata* EO, with a special emphasis on the biochemical implications of its chemical and enantiomeric compositions. However, as widely described in discussion (see Section 3), most of the major components are known for possessing interesting biological activities, some of them enantiospecific. The low availability of plant material and the consequent analytical-scale distillation prevented the authors from obtaining a pure EO, that is practically necessary to carry out a set of biological activity tests. Unless these limitations, the experimental verification of the expected bioactivities is desirable, and it could be the object of further investigations. In particular, the antioxidant capacity of *V. crenata* EO should be evaluated in a future study, as a characteristic property of many major constituents

## 5. Conclusions

Steam-distilled dried leaves of *V. crenata* produced an essential oil with a yield of 0.08% (*w*/*w*). This volatile fraction was dominated by six major compounds: germacrene D, *(E)*-β-caryophyllene, α-copaene, tricyclene, δ-cadinene, and α-pinene, which represented more than 80% of the total oil composition. The abundance of these compounds suggested that the oil may have antibacterial, antioxidant, and anti-inflammatory activities. Enantioselective analysis revealed remarkable stereochemical complexity, with the detection of several optically pure compounds, including *(1R,5R)*-(+)-α-pinene, *(1R,5R)*-(+)-sabinene, and *(S)*-(+)-β-phellandrene, which evidenced highly stereoselective biosynthetic pathways.

On the other hand, *(S)*-(-)-germacrene D, the predominant enantiomer of the main constituent, presented an enantiomeric excess of 33.4%, that could be associated with a semiochemical role in attracting insects. From a biotechnological point of view, the essential oil of *V. crenata* can be considered a natural resource of agricultural and pharmaceutical interest, given its chemical profile dominated by bioactive sesquiterpenes such as germacrene D and *(E)*-β-caryophyllene. However, the distillation yield is a limiting factor for its commercial use.

## Figures and Tables

**Table 1:** Chemical composition of *V. crenata* essential oil as determined by qualitative (GC–MS) and quantitative (GC–FID) analyses using 5% phenyl methyl polysiloxane and polyethylene glycol stationary phases. Major components (≥3.0% in at least one column) are reported in bold.

N.	Compounds		5%-Phenyl Methyl Polysiloxane						Polyethylene Glycol					Average
		RT	LRI ^a^	LRI ^b^	%	σ	Reference	RT	LRI ^a^	LRI ^b^	%	σ	Reference	%
**1**	**tricyclene**	14.57	919	921	9.4	0.76	[30]	5.56	1008	1007	9.1	1.32	[31]	**9.3**
**2**	**α-pinene**	15.51	931	932	8.2	0.62	[30]	5.95	1019	1020	8.4	0.91	[32]	**8.3**
3	α-fenchene	16.59	944	945	2.1	0.17	[30]	7.43	1059	1059	2.2	0.18	[33]	**2.2**
4	sabinene	18.67	970	969	0.1	0.01	[30]	9.27	1105	1103	0.1	0.01	[34]	**0.1**
5	β-pinene	18.83	972	974	0.1	0.01	[30]	10.13	1119	1118	0.1	0.01	[35]	**0.1**
6	myrcene	20.24	989	988	0.2	0.01	[30]	13.10	1166	1167	0.3	0.02	[36]	**0.3**
7	α-phellandrene	21.12	994	1002	0.3	0.01	[30]	12.80	1161	1160	0.2	0.01	[37]	**0.3**
8	*p*-cymene	22.73	1015	1024	0.1	0.12	[30]	20.14	1269	1268	0.1	trace	[38]	**0.1**
9	β-phellandrene	23.09	1020	1025	1.5	0.08	[30]	15.56	1204	1203	1.2	0.02	[39]	**1.6**
10	limonene	23.11	1020	1024			[30]	16.67	1196	1196	0.4	0.01	[40]	
11	benzene acetaldehyde	24.01	1032	1036	0.2	0.01	[30]	44.66	1648	1648	0.5	0.01	[41]	**0.4**
12	terpinolene	27.89	1083	1086	trace	-	[30]	20.88	1279	1280	trace	-	[42]	**trace**
13	linalool	28.86	1096	1095	trace	-	[30]		-	-	-	-	-	**trace**
14	*n*-nonanal	29.18	1100	1100	0.4	0.01	[30]	8.92	1100	1398	0.3	0.02	[43]	**0.4**
15	*(2E,4E)*-octadienal	29.71	1101	1102	trace	-	[30]	41.21	1589	1590	trace	-	[44]	**trace**
16	α-campholenal	30.68	1114	1122	trace	-	[30]	34.91	1487	1487	trace	-	[45]	**trace**
17	*trans*-pinocarveol	31.59	1127	1135	0.1	0.01	[30]		-	-	-	-	-	**0.1**
18	*trans*-verbenol	32.13	1135	1140	0.1	0.01	[30]	46.71	1684	1680	0.1	0.04	[46]	**0.1**
19	pinocarvone	33.35	1152	1160	0.1	0.01	[30]		-	-	-	-	-	**0.1**
20	*trans*-carveol	37.65	1214	1215	trace	-	[30]	55.43	1843	1849	0.1	0.01	[35]	**0.1**
21	δ-elemene	46.07	1331	1335	0.1	0.01	[30]	33.10	1459	1460	0.1	0.01	[47]	**0.1**
22	α-cubebene	46.87	1344	1348	0.1	0.06	[30]	32.47	1450	1449	trace	-	[47]	**0.1**
23	cyclosativene	47.99	1362	1369	0.4	0.20	[30]	33.61	1467	1465	0.3	0.01	[48]	**0.4**
24	ylangene	48.29	1367	1368	0.2	0.01	[37]	33.89	1472	1472	0.3	0.01	[47]	**0.3**
**25**	**α-copaene**	48.61	1374	1373	10.2	0.32	[30]	34.49	1481	1481	10.5	0.6	[47]	**10.4**
26	β-bourbonene	49.15	1381	1387	1.6	0.05	[30]	36.10	1506	1504	1.7	0.09	[47]	**1.7**
27	β-cubebene	49.53	1387	1387	0.9	0.02	[30]	37.53	1529	1527	1.0	0.39	[49]	**1.0**
28	α-gurjunene	50.77	1404	1409	0.1	0.02	[30]	36.79	1517	1519	0.2	0.01	[50]	**0.2**
**29**	***(E)*-β-caryophyllene**	51.38	1415	1417	13.3	0.4	[30]	40.91	1585	1587	13.3	0.64	[51]	**13.3**
30	β-copaene	51.98	1425	1426	0.4	0.01	[52]	40.43	1578	1580	0.4	0.01	[53]	**0.4**
31	aromadendrene	52.92	1441	1439	0.2	0.01	[30]	43.64	1631	1630	0.4	0.01	[51]	**0.3**
32	α-humulene	53.49	1451	1452	1.6	0.06	[30]	45.10	1656	1651	1.5	0.08	[54]	**1.6**
33	allo-aromadendrene	53.94	1459	1458	0.3	0.03	[30]	43.12	1622	1628	0.1	0.01	[33]	**0.2**
34	γ-muurolene	54.98	1476	1478	0.5	0.04	[30]	46.44	1679	1678	0.4	0.01	[55]	**0.5**
**35**	**germacrene D**	55.25	1481	1480	18.7	0.58	[30]	47.51	1698	1695	18.1	0.79	[56]	**18.4**
36	β-selinene	55.52	1486	1489	0.2	0.01	[30]	47.91	1705	1705	0.3	0.04	[40]	**0.3**
37	bicyclogermacrene	56.17	1497	1500	1.4	0.07	[30]	48.83	1722	1721	1.1	0.03	[57]	**1.3**
38	*trans*-β-guaiene	56.42	1501	1502	0.5	0.01	[30]	45.67	1666	-	0.7	0.73	§	**0.6**
39	*epi*-cubebol	57.28	1508	1493	trace	-	[30]	57.66	1886	1890	0.1	0.01	[58]	**0.1**
**40**	**δ-cadinene**	57.83	1517	1522	8.9	0.27	[30]	50.46	1751	1752	8.8	0.39	[59]	**8.9**
41	germacrene B	59.74	1552	1559	0.8	0.04	[30]	53.93	1815	1823	0.8	0.01	[60]	**0.8**
42	spathulenol	60.88	1572	1577	0.7	0.04	[30]	69.34	2128	2128	0.5	0.03	[61]	**0.6**
43	caryophyllene oxide	61.20	1578	1582	0.8	0.17	[30]	60.37	1939	1938	0.7	0.04	[47]	**0.8**
44	viridiflorol	62.40	1599	1592	0.8	0.03	[30]	67.29	2080	2086	0.2	0.01	[62]	**0.5**
45	unidentified (mw = 236)	69.76	1687	-	0.2	0.01	[30]	70.00	2145	-	0.5	0.04	-	**0.4**
46	*n*-heneicosane	78.23	2100	2100	1.4	0.05	[30]	68.24	2100	2100	0.5	0.03	-	**1.0**
	monoterpenes				22.0						22.1			**22.3**
	oxygenated monoterpenoids				0.3						0.2			**0.4**
	sesquiterpenes				60.4						60.0			**60.8**
	oxygenated sesquiterpenoids				2.3						1.5			**2.0**
	others				2.2						1.8			**2.2**
	total				87.2						85.6			**87.7**

N. = progressive number; RT = Retention time (min.); ^a^ calculated linear retention index (LRI); ^b^ reference linear retention index (LRI); % = percent by weight of EO; σ = standard deviation; § = identification by MS only; trace ≤ 0.1%; Average % = mean amount between the two columns. If in one column the component is trace, undetected, or sum of two peaks, only the value of the other column is reported.

**Table 2:** Enantioselective analysis of some chiral terpenes from *V. crenata* Ruiz y Pav. EO.

Chiral Selector	Enantiomer	LRI	E.D. (%)	e.e. (%)
DAC	*(1S,5S)*-(-)-α-pinene	915	-	100.0
DAC	*(1R,5R)*-(+)-α-pinene	916	100.0	
DET	*(1R,5R)*-(+)-β-pinene	949	78.3	56.6
DET	*(1S,5S)*-(-)-β-pinene	960	21.7	
DET	*(1R,5R)*-(+)-sabinene	978	100.0	100.0
DET	*(1S,5S)*-(-)-sabinene	992	-	
DET	*(R)*-(–)-α-phellandrene	1020	1.3	97.4
DET	*(S)*-(+)-α-phellandrene	1023	98.7	
DET	*(S)*-(-)-limonene	1059	6.6	86.8
DET	*(R)*-(+)-limonene	1075	93.4	
DET	*(R)*-(-)-β-phellandrene	1049	-	100.0
DET	*(S)*-(+)-β-phellandrene	1062	100.0	
DET	*(R)*-(-)-linalool	1182	49.6	0.8
DET	*(S)*-(+)-linalool	1195	50.4	
DET	*(1R,2S,6S,7S,8S)*-(-)-α-copaene	1323	62.6	25.2
DET	*(1S,2R,6R,7R,8R)*-(+)-α-copaene	1324	37.4	
DET	*(R)*-(+)-germacrene D	1460	33.3	33.4
DET	*(S)*-(-)-germacrene D	1467	66.7	

LRI = calculated linear retention index; E.D. = enantiomeric distribution (relative abundance of each enantiomer in the essential oil, EO); e.e. = enantiomeric excess, a single-value measure of enantiomeric imbalance calculated as ∣%R−%S∣ (e.g., a 60:40 mixture corresponds to 20% e.e.); DAC = 2,3-diacetyl-6-*tert*-butyldimethylsilyl-β-cyclodextrin; DET = 2,3-diethyl-6-*tert*-butyldimethylsilyl-β-cyclodextrin.

## Data Availability

The datasets presented in this article are not readily available because they are part of an ongoing study. Requests to access the datasets should be directed to the corresponding author.
